# Side Branch Occlusion After Intravascular Lithotripsy: A Case Report

**DOI:** 10.7759/cureus.89935

**Published:** 2025-08-12

**Authors:** Akito Setoguchi, Masayoshi Takeno, Saburo Kusumoto, Tatsuya Nunohiro, Koji Maemura

**Affiliations:** 1 Department of Cardiovascular Medicine, Nagasaki Harbor Medical Center, Nagasaki, JPN; 2 Department of Cardiovascular Medicine, Nagasaki University Graduate School of Biomedical Sciences, Nagasaki, JPN

**Keywords:** calcified coronary artery, drug-eluting stent (des), intravascular lithotripsy, primary percutaneous coronary intervention (pci), side branch occlusion

## Abstract

Intravascular lithotripsy (IVL) delivers acoustic shockwaves to fracture coronary calcifications and optimize stent expansion, yet side branch (SB) occlusion after IVL is rarely documented. We report the case of an 80-year-old man with prior stents in the distal right coronary and proximal left circumflex arteries who underwent elective percutaneous coronary intervention for 75% proximal left anterior descending artery (LAD) stenosis supplying four diagonal branches: the first (D1), second (D2), third (D3), and fourth (D4) diagonal branches. The instantaneous wave-free ratio was 0.82 and the fractional flow reserve was 0.77, both indicating ischemia. Optical coherence tomography (OCT) demonstrated severe, long calcification (calcification score 4; maximum arc 330°; thickness 12.1 mm; length 38.2 mm; minimum lumen area 1.46 mm²), and IVL was selected over rotational atherectomy or scoring balloon angioplasty due to high calcification score and wire bias considerations. Eight cycles of IVL with a 2.5/12 mm balloon were applied from the D4 bifurcation to proximal LAD, followed by eight cycles with a 3.0/12 mm balloon, increasing the minimum lumen area to 3.31 mm². After pre-dilation with a 2.5/13 mm scoring balloon distally and a 3.0/15 mm non-compliant balloon proximally, a 2.5/38 mm everolimus-eluting stent was implanted, and the proximal segment was post-dilated with a 3.0/15 mm non-compliant balloon. Immediately after post-dilation, the patient developed chest pain and ST-segment elevation; angiography showed new occlusion of the third diagonal branch (D3, thrombolysis in myocardial infarction (TIMI) 0) and flow reduction in the first diagonal branch (D1) from TIMI 3 to TIMI 1, while all other branches maintained TIMI 3 flow. Wire recrossing and kissing balloon inflation (1.5/10 mm semi-compliant in D3, 3.0/15 mm non-compliant in the main vessel) restored TIMI 3 flow in D3 and relieved symptoms, whereas D1 remained TIMI 1 but asymptomatic. No protective wiring had been performed initially as the affected SB was <1.5 mm, but the subsequent ischemic event indicated it should be considered a significant branch. Angiography before and after IVL showed no change suggestive of SB risk, whereas OCT performed immediately after IVL revealed new protrusion of fractured calcifications into the D1 and D3 ostia, which was considered the cause of the subsequent side branch occlusion. IVL-related SB occlusion is an extremely rare complication in the literature, but meticulous pre-procedural OCT assessment and consideration of protective wiring in high-risk bifurcation lesions may help predict and prevent this event.

## Introduction

Heavily calcified coronary lesions are a significant obstacle in percutaneous coronary intervention (PCI), often leading to device delivery failure and suboptimal stent expansion [1,2]. To address these challenges, various plaque-modification techniques such as scoring balloons, rotational atherectomy, orbital atherectomy, and excimer laser atherectomy have been developed. Plain old balloon angioplasty (POBA) dilates the lesion by exerting static radial force to compress and expand the vessel, which is generally effective for mild to moderate calcification but is inadequate for fracturing thick or extensive calcium. In contrast, debulking devices such as rotational or orbital atherectomy enlarge the coronary lumen by mechanically ablating calcified plaque, allowing treatment of heavily calcified lesions; however, their effectiveness is highly dependent on optimal wire bias, and suboptimal alignment can limit their ability to ablate the target plaque adequately [3]. In addition, their use has been associated with an increased risk of serious complications, including coronary perforation, which can be life-threatening [4].

Intravascular lithotripsy (IVL) is a novel calcium modification technology adapted from extracorporeal shock wave lithotripsy used for kidney stones. The IVL system utilizes a specialized balloon that, during low-pressure inflation, emits sonic pressure waves capable of fracturing both superficial and deep calcium while minimizing soft tissue injury [5]. This mechanism differs fundamentally from POBA and atherectomy, as it promotes vessel compliance without direct tissue removal or high-pressure trauma, and importantly, is less dependent on wire bias than atherectomy devices. Periprocedural complications of IVL are rare, with only a few cases of perforation, dissection, or no-reflow reported, and the technique has also demonstrated mid-term safety in treating severely calcified coronary lesions [6,7].

Despite these advantages, reports specifically addressing the impact of IVL on side branch (SB) patency are scarce, and its long-term outcomes remain unclear. In general, SB occlusion remains a common complication in complex bifurcation lesions with severe calcification [8,9]. For example, in the COBIS (Coronary Bifurcation Stenting) registry, Seo et al. analyzed 1,236 de novo bifurcation lesions with <75% stenosis at the SB ostium treated using a provisional stenting strategy with drug-eluting stents, and found that SB occlusion occurred in 22 of 289 lesions (approximately 7.6%) when the main vessel had moderate-to-severe calcification [10]. The paucity of IVL-related reports may reflect both the novelty of technology and the under-recognition of potential mechanisms leading to SB compromise.

We describe the case of an 80-year-old man who experienced SB occlusion after IVL in a heavily calcified bifurcation lesion, with a focus on highlighting potential mechanisms and discussing preventive strategies.

## Case presentation

**Figure 1:**
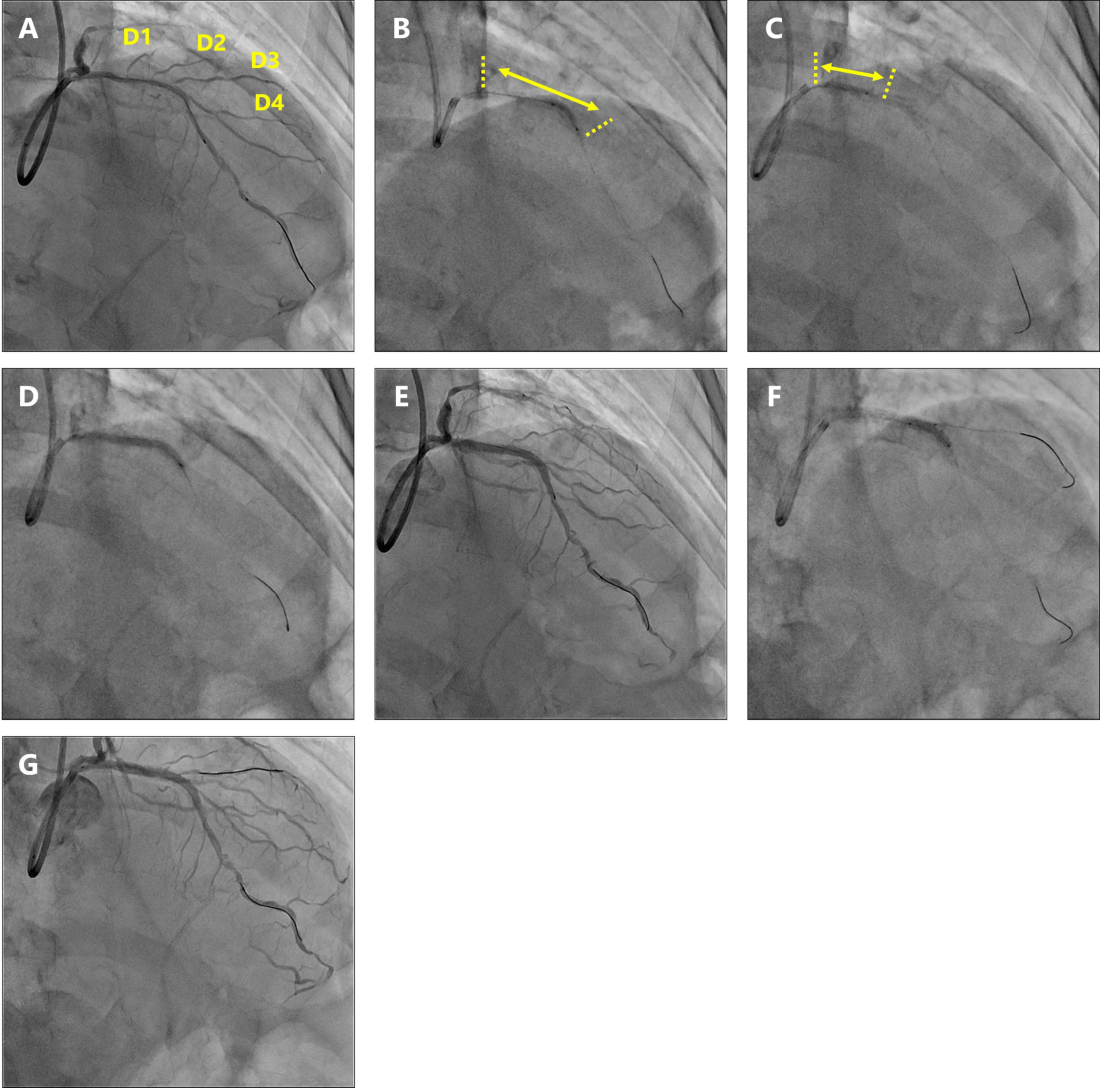
Angiographic findings A) Coronary angiography revealed 75% stenosis in both the proximal and middle left anterior descending artery. D1, 1st diagonal branch; D2, 2nd diagonal branch; D3, 3rd diagonal branch; D4, 4th diagonal branch. B) Eight cycles of IVL (2.5/12 mm catheter; Shockwave Medical, Inc., Santa Clara, California, United States) were applied from the bifurcation of D4 to the proximal LAD (yellow arrow). C) Eight cycles of IVL (3.0/12 mm catheter; Shockwave Medical, Inc.) were applied to the proximal LAD (yellow arrow). D) A 2.5/38-mm Xience Skypoint stent (Abbott Laboratories, Chicago, Illinois, United States) was deployed. E) Angiography revealed TIMI blood flow grade 1 in D1 and occlusion of D3. F) Kissing balloon inflation was performed with a 1.5/10 mm balloon in D3 and a 3.0/15 mm balloon in the main vessel. G) Final angiography showed improved flow in D3. TIMI: thrombolysis in myocardial infarction, IVL: intravascular lithotripsy, LAD: left anterior descending artery

An 80-year-old man with stents in the distal right coronary artery and proximal left circumflex artery underwent coronary angiography (CAG), revealing 75% stenosis of the left anterior descending artery (LAD). The instantaneous wave-free ratio (iFR) was 0.82, and the fractional flow reserve FFR was 0.77 (Figure 1A).

Although the patient was asymptomatic, he was admitted to our department for PCI on his own request. The patient was taking medications for hypertension, hyperlipidemia, and type 2 diabetes. On admission, his signs were as follows: body temperature, 35.1℃; blood pressure, 175/92 mmHg; heart rate (HR), 89 beats/minute; and peripheral oxygen saturation, 97% in room air. Laboratory tests showed mild hyperglycemia with elevated fasting plasma glucose (206 mg/dL) and HbA1c (6.7%), impaired renal function evidenced by reduced estimated glomerular filtration rate (42.1 mL/minute/1.73 m²), creatinine (1.28 mg/dL), and blood urea nitrogen (24.3 mg/dL). Liver enzymes were slightly elevated (aspartate aminotransferase 33 U/L, alanine aminotransferase 45 U/L), and N-terminal pro-brain natriuretic peptide was mildly increased (165 pg/mL). Hematological parameters were within normal limits (Table 1).

**Table 1:** Laboratory findings on admission WBC: white blood cell count, RBC: red blood cell count, Hb: hemoglobin, PLT: platelet count, TG: triglyceride, LDL-C: low-density lipoprotein cholesterol, HDL-C: high-density lipoprotein cholesterol, FPG: fasting plasma glucose, HbA1c: glycated hemoglobin, TP: total protein, AST: aspartate aminotransferase, ALT: alanine amino transferase, CK: creatine kinase, BUN: blood urea nitrogen, Cre: creatinine, eGFR: estimated glomerular filtration rate, CRP: C-reactive protein, NT-pro BNP: N terminal-pro brain natriuretic peptide.

Parameters	Patient Values	Units	Reference Ranges
WBC	5.1	×10³/μL	3.3-8.6
RBC	4.52	×10⁶/μL	4.35-5.55
Hb	14.2	g/dL	13.7-16.8
PLT	198	×10³/μL	158-348
TG	227	mg/dL	40-234
LDL-C	59	mg/dL	65-163
HDL-C	43	mg/dL	38-90
FPG	206	mg/dL	73-109
HbA1c	6.7	%	4.9-6.0
TP	7.6	g/dL	6.6-8.0
AST	33	U/L	13-30
ALT	45	U/L	10-42
CK	157	U/L	59-248
BUN	24.3	mg/dL	8.0-20.0
Cr	1.28	mg/dL	0.65-1.07
eGFR	42.1	mL/min/1.73m²	>90
CRP	0.34	mg/dL	0.00-0.14
Na	139	mEq/L	138-145
K	4.9	mEq/L	3.6-4.8
Cl	102	mEq/L	101-108
NT-proBNP	165	pg/mL	<125

Twelve-lead electrocardiography (ECG) showed HR, 80 beats/minute; sinus rhythm; left axis deviation; and flat T waves in aVL. Transthoracic echocardiography showed left ventricular diastolic diameter, 37 mm; left ventricular end-systolic diameter, 22 mm; and ejection fraction, 73% with no asynergy.

**Figure 2:**
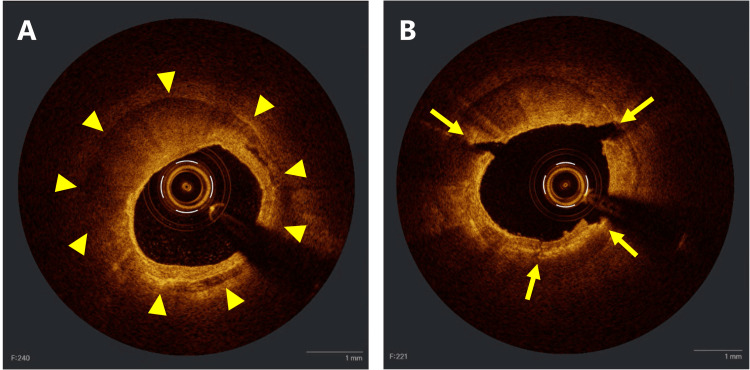
OCT findings before and after IVL A) The coronary artery exhibited severe calcification (yellow arrowheads). The calcification score is 4 (maximum calcification angle: 330°; maximum calcification thickness, 12.1 mm; calcification length, 38.2 mm). B) After IVL, multiple fractures in the calcification were evident (yellow arrows). IVL: intravascular lithotripsy, OCT: optical coherence tomography

Optical coherence tomography (OCT) showed a minimum lumen area (MLA) of 1.46 mm² and severe calcification (calcification score 4; maximum calcification angle 330°; maximum calcification thickness 12.1 mm; calcification length 38.2 mm), indicating that conventional balloon dilatation would likely be insufficient to achieve adequate expansion, thus justifying the use of IVL (Figure 2A).

**Figure 3:**
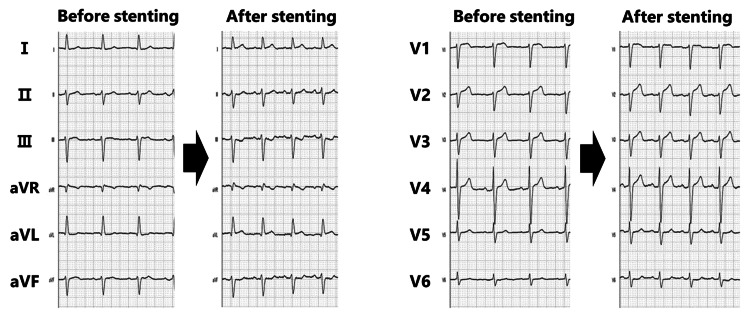
Twelve-lead electrocardiogram After stent placement, ST elevation was observed in leads I and aVL, and ST depression was evident in leads II, III, and aVF.

Eight cycles of IVL (2.5/12 mm catheter; Shockwave Medical, Inc., Santa Clara, California, United States) were applied from the bifurcation of the fourth diagonal branch (D4) to the proximal LAD, followed by eight cycles of IVL (3.0/12 mm catheter; Shockwave Medical, Inc.) to the proximal LAD (Figure 1B, 1C). Fracturing of the calcification and enlargement of the MLA to 3.31 mm^2^ were observed on OCT (Figure 2B). Pre-dilation was performed using a 2.5/13 mm scoring balloon for the distal side and a 3.0/15 mm non-compliant balloon for the proximal side. After confirming expansion on OCT, a 2.5/38 mm everolimus-eluting stent was deployed (Figure 1D). After the proximal part of the stent was post-dilated with a 3.0/15 mm non-compliant balloon, the patient complained of chest discomfort, and the ECG showed ST elevation (Figure 3).

**Figure 4:**
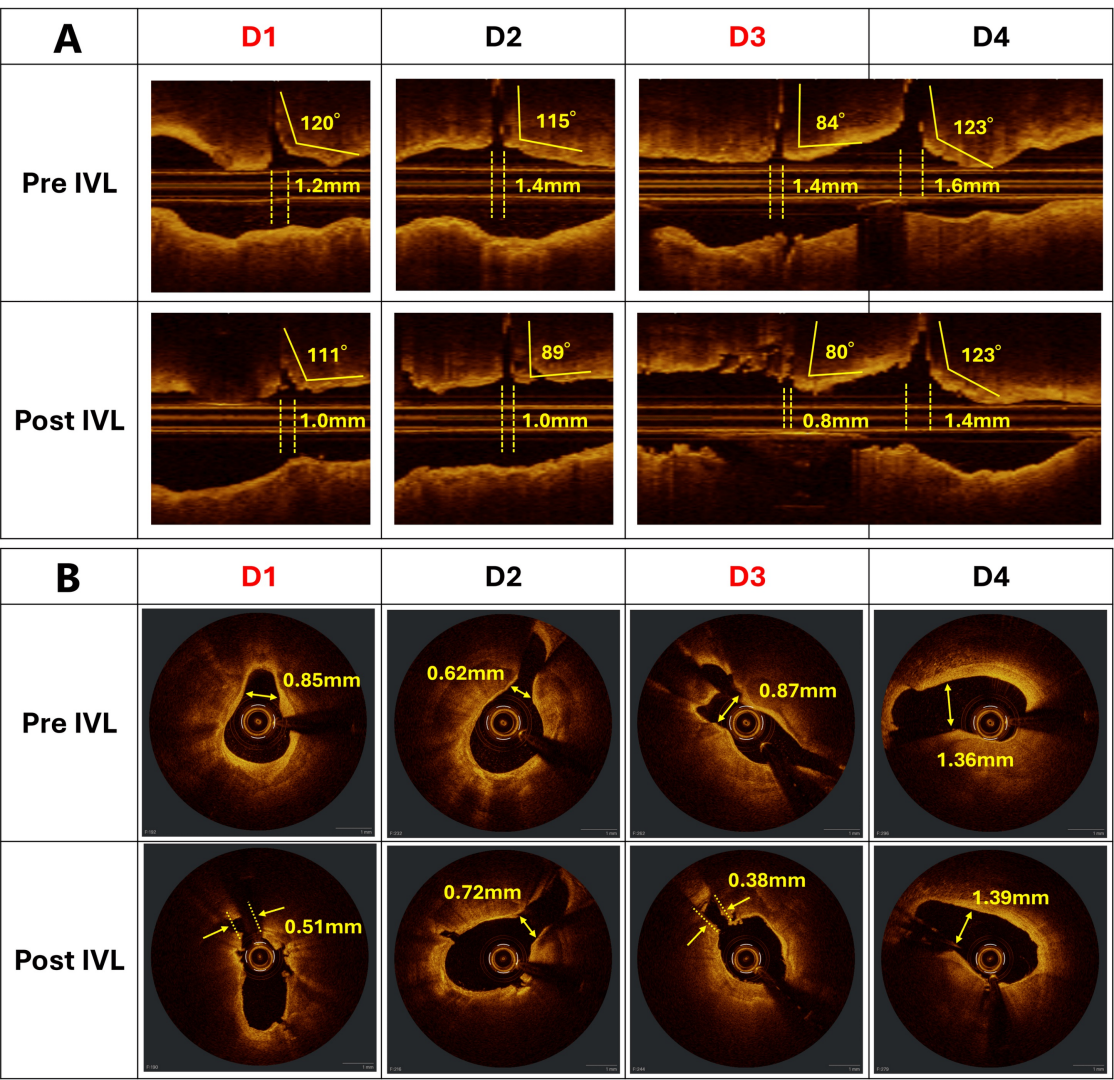
OCT findings before and after IVL A) On longitudinal OCT images before and after IVL, solid lines indicate the CT angles of side branches (D1–D4), and dashed lines indicate BP-CT lengths. Numerical values are provided in the main text. B) On cross-sectional images, D1 and D3 showed narrowing at the entry point due to fractured calcifications post-IVL. Yellow arrows indicate the width of the side branches before and after IVL (D1: 0.85 → 0.51 mm, D2: 0.62 → 0.72 mm, D3: 0.87 → 0.38 mm, D4: 1.36 → 1.39 mm), highlighting significant narrowing in D1 and D3. IVL: intravascular lithotripsy, OCT: optical coherence tomography

Angiography revealed sub-occlusion of the first diagonal branch (D1) and occlusion of the third diagonal branch (D3) (Figure 1E). A wire was passed through D3, and kissing balloon inflation was performed with a 1.5/10 mm semi-compliant balloon in D3 and a 3.0/15 mm non-compliant balloon in the main vessel (Figure 1F). The final angiography showed improved flow in D3, with resolution of chest symptoms and ECG changes (Figure 1G). All branches except D1 maintained TIMI grade 3 flow, whereas D1 showed a reduction from grade 3 to grade 1. However, as the patient remained asymptomatic and no electrocardiographic changes were observed, no additional intervention was undertaken. Postoperative OCT revealed that the ostia of D1 and D3 had been narrowed by fractured calcifications, impairing flow (Figure 4).

After PCI, continuous intravenous infusion of unfractionated heparin and nicorandil was administered for 24 hours. Creatine kinase peaked at 415 U/L at 13 hours after PCI and subsequently showed a declining trend (Table 2).

**Table 2:** Laboratory data at admission and at each time point after PCI “Post-PCI (Xh)” indicates X hours after the completion of PCI. AST: aspartate aminotransferase, ALT: alanine aminotransferase, CK: creatine kinase, hs-TnT: high-sensitivity troponin T, PCI: percutaneous coronary intervention

Parameters	Admission	Post-PCI (0h)	Post-PCI (1h)	Post-PCI (5h)	Post-PCI (9h)	Post-PCI (13h)	Post-PCI (17h)	Post-PCI (21h)	Units	Normal ranges
AST	33	32	47	45	47	50	47	40	U/L	13-30
ALT	45	44	44	38	35	37	35	29	U/L	10-42
CK	157	112	370	371	381	415	394	315	U/L	59-248
hs-TnT	–	0.044	–	–	–	1.030	–	–	ng/mL	<0.018

As the patient remained asymptomatic and the laboratory markers improved, he was discharged on postoperative day 2. Dual antiplatelet therapy with prasugrel (3.75 mg/day) and aspirin (100 mg/day) was continued for three months after discharge, followed by prasugrel monotherapy. Routine follow-up coronary angiography was not performed owing to the patient’s advanced age; however, he remained cardiovascular event-free during the one-year follow-up period.

## Discussion

Previous studies have reported some angiographic predictors of developing stenosis or occlusion of SBs while treating the main branch [9,11]. The RESOLVE (Risk Prediction of Side Branch Occlusion in Coronary Bifurcation Intervention) score, an angiographic risk stratification tool, can help identify patients at risk for SB occlusion during bifurcation intervention [12]. Other OCT findings include "calcified lesions of the main vessel," "small bifurcation angle of the side branch," and "prominent, long carina" [8,11].

The mechanisms by which calcification at the bifurcation causes SB occlusion include the need for high pressure during balloon dilation and stent deployment, and reduced compliance of the wall opposite the SB, increasing the risk of carina shift by the balloon [12-14]. Proper management of calcified bifurcation lesions is thus crucial. Rotational atherectomy of the main vessel is associated with a reduced incidence of SB stenosis; it should be performed in cases showing severe calcification [15,16].

This report describes a patient with a calcified bifurcation lesion treated with IVL. The patient’s pre-procedural angiography showed ≥50% stenosis in the proximal segments of D1-3, with RESOLVE scores of 9 (intermediate risk) for D1-3 and 6 (low risk) for D4, and no changes were observed between before and after IVL. It is likely that the absence of change in the score reflects the fact that the calcium fractures induced by IVL were too subtle to be detected by angiographic assessment. As vessels with a diameter of <1.5 mm are generally unlikely to cause clinical issues, routine SB protection is not recommended [17]. However, in the present case, occlusion of D3 led to symptoms and ECG changes, indicating that appropriate SB protection should have been performed. This aligns with the criteria of the European Bifurcation Club for a significant SB, as "a branch that one does not want to lose in the global context of a particular patient (symptoms, location of ischemia, branch responsible for symptoms or ischemia, viability, collateralizing vessel, left ventricular function, etc.)" [18].

For lesions with an SB entry diameter exceeding 1.5 mm, OCT-based risk factors for SB occlusion include a carina tip (CT) angle less than 50° and a length from the proximal branching point to the carina tip (BP-CT length) less than 1.7 mm [19]. In this case, CT angles before IVL were: D1, 120°; 2nd diagonal branch (D2), 105°; D3, 84°; and D4, 123°. No significant changes were apparent after IVL (D1, 111°; D2, 89°; D3, 80°; D4, 123°). BP-CT lengths before IVL were: D1, 1.2 mm; D2, 1.4 mm; D3, 1.4 mm; and D4, 1.6 mm. After IVL, these lengths were: D1, 1.0 mm; D2, 1.0 mm; D3, 0.8 mm; and D4, 1.4 mm after IVL, showing a significant change in the occluded D3 (Fig. 4A). However, for lesions with SB entry diameters <1.5 mm, the applicability of these cutoff values remains unclear, highlighting the need for further evaluation using imaging modalities such as OCT.

In the present case, fractured calcifications observed on OCT images immediately after IVL visibly narrowed the SBs. In addition to the protrusion of fractured calcified fragments, plaque redistribution toward the SB ostium could also theoretically contribute to SB compromise after IVL; however, OCT findings indicated that calcium displacement was the predominant mechanism. In Japan, stent implantation is generally performed following IVL treatment. Compared with conventional plain old balloon angioplasty (POBA), IVL induces multiple deep and fine calcium fractures [20]. These fractures may destabilize calcified plaques and promote plaque shift toward the ostium of SBs, thereby increasing the risk of SB occlusion after stent deployment. Therefore, a detailed OCT assessment after IVL is essential to predict potential SB impairment. When high-risk plaque morphology or heavy calcification near SBs is identified on OCT, and wire bias is not problematic, additional rotational atherectomy of the main vessel or SB protection should be considered. Even in small vessels with a diameter of <1.5 mm, as in the present case, ischemic symptoms may occur; thus, if post-IVL OCT reveals structural changes such as narrowing at the SB ostium, timely preventive measures-such as wiring or balloon dilatation-should be undertaken to avoid SB compromise. Further accumulation of clinical cases and focused research is warranted to elucidate the relationship between IVL and SB occlusion.

## Conclusions

IVL is effective and safe for treating calcified lesions; however, its impact on SB occlusion remains poorly understood. When pre-stenting OCT shows high-risk features-such as fractured calcification adjacent to the SB or shortened BP-CT length-prophylactic protection should be considered for clinically significant SBs. Because unprotected SB occlusion may cause ischemia or infarction, preventive strategies are important. As this is a single case, the findings should be interpreted with caution, and further research and registry data are needed to validate OCT-based risk stratification in IVL cases.

## References

[1] Bourantas CV, Zhang YJ, Garg S (2014). Prognostic implications of coronary calcification in patients with obstructive coronary artery disease treated by percutaneous coronary intervention: a patient-level pooled analysis of 7 contemporary stent trials. Heart.

[2] Généreux P, Redfors B, Witzenbichler B (2017). Two-year outcomes after percutaneous coronary intervention of calcified lesions with drug-eluting stents. Int J Cardiol.

[3] Oda T, Kinoshita Y, Miyahara M, Maekawa Y, Nishikawa H, Suzuki T (2024). Wire bias modification with reverse orbital atherectomy for safer rotational atherectomy in calcified bifurcation. J Cardiol Cases.

[4] Protty MB, Hussain HI, Gallagher S (2021). Rotational atherectomy complicated by coronary perforation is associated with poor outcomes: analysis of 10,980 cases from the British Cardiovascular Intervention Society database. Cardiovasc Revasc Med.

[5] Ali ZA, Nef H, Escaned J (2019). Safety and effectiveness of coronary intravascular lithotripsy for treatment of severely calcified coronary stenoses: the Disrupt CAD II study. Circ Cardiovasc Interv.

[6] Saito S, Yamazaki S, Takahashi A (2023). Intravascular lithotripsy for vessel preparation in calcified coronary arteries prior to stent placement　- Japanese Disrupt CAD IV study 2-year results. Circ Rep.

[7] Sagris M, Ktenopoulos N, Dimitriadis K (2024). Efficacy of intravascular lithotripsy (IVL) in coronary stenosis with severe calcification: a multicenter systematic review and meta-analysis. Catheter Cardiovasc Interv.

[8] Fujino Y, Attizzani GF, Tahara S (2014). Impact of main-branch calcified plaque on side-branch stenosis in bifurcation stenting: an optical coherence tomography study. Int J Cardiol.

[9] Bai J, Yue Y, Feng HQ (2019). Impact of main vessel calcification on procedural and clinical outcomes of bifurcation lesion undergoing provisional single-stenting intervention: a multicenter, prospective, observational study. J Geriatr Cardiol.

[10] Seo JB, Shin DH, Park KW (2016). Predictors for side branch failure during provisional strategy of coronary intervention for bifurcation lesions (from the Korean Bifurcation Registry). Am J Cardiol.

[11] Kini AS, Vengrenyuk Y, Pena J (2017). Plaque morphology predictors of side branch occlusion after provisional stenting in coronary bifurcation lesion: results of optical coherence tomography bifurcation study (ORBID). Catheter Cardiovasc Interv.

[12] Secco GG, Buettner A, Parisi R (2019). Clinical experience with very high-pressure dilatation for resistant coronary lesions. Cardiovasc Revasc Med.

[13] Pescetelli I, Zimarino M, Ghirarduzzi A, De Caterina R (2015). Localizing factors in atherosclerosis. J Cardiovasc Med (Hagerstown).

[14] Allali A, Abdel-Wahab M, Traboulsi H (2020). Impact of lesion preparation technique on side branch compromise in calcified coronary bifurcations: a subgroup analysis of the PREPARE-CALC trial. J Interv Cardiol.

[15] Sturm R, Armstrong EJ, Benhuri B (2021). Orbital atherectomy for treatment of severely calcified coronary artery bifurcation lesions: a multicenter analysis. Cardiovasc Revasc Med.

[16] Mizuno Y, Sakakura K, Jinnouchi H (2022). Impact of rotational atherectomy on the incidence of side branch compromise in calcified bifurcation lesions undergoing elective percutaneous coronary intervention. J Cardiol.

[17] Peng XF, Huang JB, Xing ZH (2018). Small side branch compromise related to main vessel stenting: a retrospective cohort study comparing different treatment strategies. Medicine (Baltimore).

[18] Louvard Y, Medina A (2015). Definitions and classifications of bifurcation lesions and treatment. EuroIntervention.

[19] Watanabe M, Uemura S, Sugawara Y (2014). Side branch complication after a single-stent crossover technique: prediction with frequency domain optical coherence tomography. Coron Artery Dis.

[20] Kawai K, Sato Y, Hokama JY (2023). Histology, OCT, and micro-CT evaluation of coronary calcification treated with intravascular lithotripsy: atherosclerotic cadaver study. JACC Cardiovasc Interv.

